# Higher-Order Executive Function in Middle School: Training Teachers to Enhance Cognition in Young Adolescents

**DOI:** 10.3389/fpsyg.2022.867264

**Published:** 2022-05-03

**Authors:** Jacquelyn F. Gamino, Courtney Frost, Russell Riddle, Janet Koslovsky, Sandra B. Chapman

**Affiliations:** The University of Texas at Dallas, Center for BrainHealth, Richardson, TX, United States

**Keywords:** cognitive training, higher-order executive function, far transfer, educator training, educator professional development

## Abstract

The epoch of adolescent brain development is an ideal time to train complex thinking skills, and middle schools provide an ideal environment to train and foster this acquisition. Unfortunately, few teachers are equipped with enough knowledge of the science of learning and evidence-based methodology, to ensure all students are given sufficient opportunity to develop their cognitive capacity to the fullest. Using our evidenced-based higher-order executive function training program, we trained current teachers to provide cognitive training to their students. The results of this study demonstrate the efficacy of teacher-implemented intervention for immediate improvement in high-level executive function capacities such as gist-reasoning and interpretive statement production. More importantly, we found evidence of far transfer *via* students’ improved academic performance in all standardized test content areas (Reading, Mathematics, Science, and Social Studies) when compared to their untrained peers. Our findings support the importance of providing intensive professional development that afford educators with a greater understanding of the brain, how we learn, and the importance of evidence-based programs to advance and instill high-level executive function in all students.

## Introduction

The neurobiology of learning, and in particular the core concept of neuroplasticity, have the potential to directly transform education-based professional development and affect how teachers and students think about their own learning (Dubinsky et al., 2013). Neuroscience provides a biological basis for how learning occurs in the brain, and as such, should be utilized to mold learning theories (Meltzoff et al., 2009; Guerriero, 2017) as the scientific evidence presents concrete and fundamental contributions to the knowledge of learning processes and individual student characteristics (Voss et al., 2011). Continuing education and professional development trainings with an emphasis on neuroscience, executive function, and learning processes, have been shown to increase teacher content knowledge and improve student-centered teaching methods (MacNabb et al., 2006; Roehrig et al., 2012). Notably, research suggests teachers are eager to learn about the brain and its role in learning, but desire neuroscience instruction explained in an accessible and easily applied manner (Tham et al., 2019). Although it is challenging to formulate a plan of action for the integration of neuroscience in educational practices, through collaborative efforts classroom teaching strategies have been shown to foster student success (Dekker et al., 2012; Privitera, 2021).

Improving teacher education is the best investment we can make to create a better future; however, it is also one of the most difficult initiatives to navigate. Teachers report frustration when they cannot connect to their students and create positive academic growth (Brick et al., 2021a). Teachers, who consistently endure high levels of stress without adequate training and support, often abandon the profession. Poor training may account for the United States’ educator attrition data that disclose 1/3 of teachers leave the field within the first 5 years of their careers (Musset, 2010). If teachers feel supported and are given opportunities to learn promising evidence-based methods, we can improve retention of teachers. Garet et al. (2001) reported that providing teachers with “hands-on” professional learning enhances their knowledge and ability to teach their content. Trainings that incorporate modeling and allow teachers to practice and reflect on new strategies help educators consider the learning experience and the application of new information. When teachers experience executive function demands similar to those placed on their students, they can better anticipate their students’ frustrations and present strategies to help relieve cognitive overload and the resulting academic pressure (Wei et al., 2009; Diamond and Ling, 2020). The impact of professional development needs to go beyond a temporary one-time boost and address real-life demands across all school subject matter. As such, it is critical to embed evidence-based application of executive function into the foundation of instruction (Chapman et al., 2012). When presenting teachers with valuable brain science education, a focus on executive function is essential for understanding the learning processes (Diamond, 2012). Executive function refers to the range of controlled mental processes such as inhibition and selective attention, as well as the constructs of working memory, and cognitive flexibility. The dissociated component structure suggested by Diamond (2006) proposes that these fundamental domains have different developmental trajectories, reaffirming the importance of instilling executive function abilities during school age. Similarly, the “unity and diversity” framework of Miyake et al. (2000) focuses on inhibition, information updating and monitoring, and shifting as interrelated but distinct components, providing significant evidence for middle childhood and early adolescent executive function development (Miyake et al., 2000; Best and Miller, 2010).

Although children are not born with these skills, they have the innate potential to develop and strengthen executive function regardless of their home circumstances (Diamond and Lee, 2011). Basic executive function abilities such as selective focus and suppression of impulses are necessary for successful learning, but they are not sufficient by themselves. Higher-order executive functions that encompass reasoning, problem-solving, and flexible thinking become necessary as demands of academic and social experiences increase. Infusing executive function skills in education ultimately improves real-life goal attainment, particularly in the area of academic achievement (Chapman et al., 2012; Jacob and Parkinson, 2015; Diamond and Ling, 2020; Smid et al., 2020). Mounting evidence for the trainability of executive functions provides schools with a significant responsibility and valuable opportunity (Diamond and Lee, 2011; Diamond and Ling, 2020)—if educators understand the importance of these skills. Executive functions are not only related to higher-level cognitive abilities that contribute to academic success and confidence in classroom performance, but also generalize to build stronger social skills and emotional regulation (Titz and Karbach, 2014).

Executive function has become a trending buzzword in education when discussing the importance of skills, such as goal setting, planning, and critical thinking; however, there is a dearth of scientific evidence-based interventions in schools. Despite the trend to talk about executive function, educators’ limited knowledge and understanding of the nature and importance of executive function for learning may prevent the acceptance, development, and effective implementation of interventions that contribute to all students’ success (Morgan-Borkowsky, 2012). Teachers need to understand how to consider the executive function components that might mitigate students’ difficulty acquiring a particular subject matter. Neuroscience informs us that well-developed executive function is imperative to leveling and elevating the playing field for all students across the United States, as children with better executive function engage more effectively with classroom learning activities (Moffitt et al., 2011; Luby et al., 2013). Evidence indicates that executive function mediates the relationship between compromised home environments and academic achievement (Diamond, 2012).

Increasing evidence supports the importance of early-life conditions to the development of children’s cognitive processing (Hackman and Farah, 2009; Hackman et al., 2010, 2015). As such, evidence-based executive function practices and trainings have the potential to reduce achievement gaps associated with poverty (Kavanaugh et al., 2019), and change the trajectory of children from low socio-economic status families who often demonstrate lower levels of executive function (Moffitt et al., 2011; Luby et al., 2013; Gamino et al., 2014). Mounting evidence suggests well-developed executive function is imperative for future success in all domains (Chapman and Mudar, 2014).

Imal and Wexler (2018) demonstrated that executive function training is beneficial to learning and increasing “school readiness.” The researchers found weekly brain training implemented across a 4-month period in kindergarteners significantly increased student gains on school-administered achievement tests in first grade. These findings illustrate the relationship between cognitive skill development and the far-transfer of skills that foster academic achievement (Wexler et al., 2016; Imal and Wexler, 2018).

Furthermore, Wexler et al. (2016) found executive function training made a significant difference regardless of student IQ, and when compared to other school interventions, (e.g., tutoring) produced higher achievement in mathematics. These studies highlight the importance of providing executive function enhancement to entire classrooms of children *via* teacher training that embraces evidence-based education practices to achieve universal student growth at all developmental stages.

A key aspect of executive function development is cognitive flexibility, the ability to shift between different tasks or goals, that allows one to embrace multiple perspectives and develop new solutions to everyday problems (Karbach and Unger, 2014). Cognitive flexibility reflects the ability of the brain to adapt and make changes to thought processes using environmental cues and/or new information to appropriately meet the needs of the situation, assimilate and consider various perspectives, and change course when an initial pathway is not working. This construct is employed regularly to create an essential connection between cognitive function and situational experience, whether academic, social, or personal. Cognitive flexibility is key for a growth mindset and a predictor of academic achievement particularly for low-income students (Claro et al., 2016). Establishing a solid foundation in these skills provides individuals the ability to regulate and adapt their thoughts and actions to novel situations (Miyake et al., 2000). Thus, cognitive flexibility allows for divergent thinking in a continuously changing environment and provides increased capabilities to integrate contextual information from ones’ personal experiences.

Teachers who are provided avenues to recognize, understand, encourage, and train cognitive flexibility and creative thinking are better equipped to help students construct a neural foundation capable of handling unpredicted changes in ones’ environments. Well-developed cognitive flexibility produces superior decision-making abilities in students and openness to tackle difficult content (Blackwell et al., 2007; Steinbeis and Crone, 2016). In other words, cognitive flexibility directly supports persistence (Steinbeis and Crone, 2016). Behavioral and neuroimaging results demonstrate the rapid increases of cognitive flexibility during early and middle adolescence, suggesting children are highly sensitive to developmental and environmental experiences during the middle school years (Buttelmann and Karbach, 2017).

Although teachers have an abundance of trainings and professional development opportunities available to them, historically there has been little evidence these trainings create long-term benefits in students (Wei et al., 2009). Alternatively, research demonstrates that neuroscience concepts can be used to *directly* improve teachers’ understanding of student learning and teachers’ need to increase metacognition (Blackwell et al., 2007). Recent evidence suggests that professional developments that focus on neuroscience have the capacity to build education self-efficacy, motivation, and personal responsibility (Brick et al., 2021b). Chang and colleagues demonstrated that teachers who participated in a graduate-level professional development course that explored educational neuroscience concepts, shifted their focus from content and classroom management issues to their students’ needs, recognizing the importance of their students’ experiences and desire to be agents of their own learning (Chang et al., 2021).

Despite the importance of professional development, it is universally undervalued. A focus on improving teachers and teaching practices is crucial for achieving a higher-quality student education [Organisation for Economic Cooperation and Development (OECD), 2005]. Educational programs and policies that do not translate ideas into tangible methodology are likely to be ineffective. Likewise, there is an expectation that teaching methods will automatically nurture higher-order thinking skills and strengthen student performance despite limited knowledge of cognitive neuroscience findings (OECD, 1998). Instilling the ideals of higher-order thinking requires teachers who understand learning processes to reinforce and instill connections between students’ experiences and curriculum goals (Darling-Hammond et al., 2017). Data indicate that the intensity and duration of professional development offered to US teachers is not at the level research suggests is necessary to have a noticeable impact on instruction and student learning (National Center of Educational Statistics, 2019). Comparisons of American teachers’ participation in professional development with that of teachers in the international community demonstrate that the United States is substantially behind other nations in providing the kinds of powerful professional learning opportunities that are more likely to build teaching capacity and have significant impact on student learning (National Staff Development Council, 2019). While there is no known evidence regarding the amount of training required to help teachers assimilate new ideas into their day-to-day instruction, it is generally found that brief introductions to new concepts do not provide the impetus to apply the newly learned information. In order to transform teaching, professional development must create opportunities for active intensive learning rather than simply layering new strategies on top of the old (Garet et al., 2001).

To ensure teachers are receiving accurate information about the importance of the brain and learning, teachers must first learn truths. “Neuromyths” or “misconceptions generated by a misunderstanding, a misreading, or a misquoting of facts” create an obstacle for providing teachers with the best teaching tools (Organisation for Economic Co-operation, and Development, 2002). Many teachers, even those with a passion for science and brain research, demonstrate significant levels of accord with neuromyths, suggesting the difficulty of distinguishing science-based facts from popular beliefs. For example, a commonly embraced misconception in education is the suggestion that people are “left or right brained” suggesting that both hemispheres do not contribute harmoniously to enable learning (Dekker et al., 2012). Explicit evidence-based education for teachers is necessary to reduce the proliferation of, and acquiescence to, neuromyths (Dekker et al., 2012). Most teachers regularly read and research new ideas to incorporate into their classrooms, but without an evidence-based framework for these programs, interventions often bring uncertainty or have negligible results. Providing teachers with tools to integrate evidence-based executive function training into everyday instruction has the potential to increase positive outcomes for students and inform scalability. Research regarding healthy brain development and neuroplasticity inform evidence-based, targeted interventions that address the growing crisis of impoverished children (Diamond and Ling, 2016; Jensen et al., 2017). The question remains, “How well are these interventions implemented in school settings?”

We previously pioneered higher-order executive function training (EF training) in classrooms using the Strategic Memory Advanced Reasoning Training (*SMART©*; Chapman and Gamino, 2008) program. Our evidence found SMART strengthened adolescent higher-order executive function (Gamino et al., 2010, 2014). We did not know if our research findings would equivocally translate into teacher-guided implementation. The next logical research undertaking was to determine if disseminating the cognitive training to middle school educators would produce similar results. Scaling the EF training program to enable educator implementation required developing an educator training protocol and addressing potential fidelity issues.

The aim of the current study was twofold. First, we wanted to determine if cognitive training delivered by educators during regular classes would significantly change students’ ability to process information at a deeper level, commensurate with the changes demonstrated by students in our former researcher-led studies (Gamino et al., 2010, 2014). Second, we examined whether students who received cognitive training from their teacher demonstrated far transfer of EF training by exhibiting improved academic performance as determined by state-mandated standardized tests.

We hypothesized that teachers could be trained to successfully implement EF training to demonstrate increases in higher-order executive function at post-test. Additionally, we hypothesized that students would demonstrate far-transfer effects through their academic performance on state mandated testing.

## Materials and Methods

### Teacher-Trainees

After detailed communication and planning with district and school administration, an urban public middle school on the southern border of Texas agreed to participate. The school’s principal recruited three teachers to attend our professional development and subsequently implement our EF training program in their classrooms during regular school hours.

For this study, three of the school’s four eighth grade English Language Arts (ELA) teachers attended our 5-day summer workshop at The University of Texas at Dallas’ Center for BrainHealth. The teachers who participated agreed to implement our EF training program in their classes during the subsequent fall semester. The one remaining eighth grade ELA teacher was given the choice of participating, but ultimately decided not to attend the professional development nor implement the EF training program in her classroom.

### Participants

The sample for this study included the eighth grade students who were pre-assigned by the school scheduling administrator to take English Language Arts from one of the three participating ELA teachers. The participants ranged in age from 13 to 15 years, the mean age was 13.52. Combined, the three participating teachers’ ELA classrooms (one teacher had four class periods of students per day and the other two teachers had six periods of students per day) contained 315 students who received the EF training program, representing 71% of the entire eighth grade. The ecological validity of this study was maintained by inviting all students who were enrolled in three teachers’ ELA classes to participate in the study, without exclusion for neurodevelopmental differences. Hence, our data reflect the typical sociodemographic of the school.

The EF training program was adopted in the three classrooms as part of the regular school curriculum; thus, the number of participants in the training differed from the number of students who individually assented to participate in the optional cognitive testing. No individual, personal data regarding neurodevelopmental disorders or IQ were collected from the participants. School administrators provided de-identified outcomes for the State of Texas Assessment of Academic Readiness (STAAR) for the group of 315 trained students at the end of the school year. Of the 315 students who were enrolled in the participating ELA classes and received EF training, 306 were in attendance on the day of the Scale of Advanced Reasoning (SOAR©, Chapman et al., 2006) baseline assessment. Of the 306 students in the participating classes, 265 (133 females) consented to share their pre-training data in accordance with Institutional Review Board guidance, while 41 students (15 females) declined. On the day of the post-training assessment, 308 students were in attendance, of whom 251 (123 females) gave their consent while 57 (22 females) declined. No significant differences were found in the proportion of males to females who declined participation. Ultimately, 213 students consented to share their pre- and post-training data obtained from the SOAR.

Male and female participants were equally represented and 93.4% of the participants identified as Hispanic descent. All classes were taught in English, as the school promotes using English in the classrooms to ensure students are well versed in the primary language of the United States. Overall, the school student body was reflected as 99% Hispanic. Additionally, 98% of the student body met criteria for “economically disadvantaged” designation.

### Quasi Control Group

The students in the non-participatory eighth grade teacher’s ELA classes were ethnically and socioeconomically comparable to the participants. Thus, we used the publicly available state-mandated standardized test data to compare performance levels between groups before and after the experimental group underwent EF training.

### Executive Function Training

#### Strategic Memory Advanced Reasoning Training

The cognitive training program used in the present research was the Strategic Memory Advanced Reasoning Training (SMART©, Chapman and Gamino, 2008) program developed at The University of Texas at Dallas’ Center for BrainHealth. The manualized higher-order executive function training program consisted of 10, 45-min classroom sessions that were delivered over a 1-month period. The program instructed students in metacognitive strategies and practice that fostered top-down processing *via* abstraction of meanings from information (Gamino et al., 2010). The EF training program presented hierarchical cognitive processes through interactive group exercises and pen and paper practice in student instructional manuals. The guided instruction emphasized the integration of world knowledge with important facts in order to capture overarching themes and facilitate gist-reasoning (Mayer, 1989; Chapman et al., 2006; Gamino et al., 2010). The EF training program engaged students in classroom-wide discussions encouraging verbal and written expression of ideas and thought processes facilitated by the instructor’s presentation of the program’s open-ended questions.

Specifically, the EF training program focused on: (1) deliberate inhibition of extraneous information, (2) chunking and organizing relevant information, (3) inferencing, (4) paraphrasing, (5) synthesis of important details, (6) interpretations or take-home messages that could be generalized to broader contexts, and (7) abstraction of deeper meanings (see Addendum). The crux of our EF training program was to engage students in the practice of thinking about information at a level beyond the “black and white” of details specifically stated. In other words, the EF training practices underscored deliberate analysis and interpretation, allowing students the creative freedom to think about information on their own terms.

During the first five EF training sessions, students were specifically taught metacognitive processes that form a foundation for abstracting meaning from information. Students practiced basic executive function that enabled selective attention, planning, organization, chunking of important facts, inferential and interpretive paraphrasing, and synthesis of details. After basic executive function processes were introduced, practiced, and honed, the EF training program utilized the foundation provided by metacognitive awareness of basic executive function to instill top-down processing.

During the last five EF training sessions, the teacher-led program provided students with practice synthesizing world knowledge with new information to promote depth of understanding. Through pointed questioning and carefully constructed explanations that focused on cognition, students used high-level executive function to analyze, explore different perspectives, reason through unstated meanings, and create new knowledge through innovative thought. During the final sessions of EF training, students practiced synthesizing the aforementioned processes to foster top-down thinking skills. In other words, students were guided to deliberately contemplate meanings at the onset of their exposure to new information.

The texts and materials used within the program to practice the cognitive processes were similar to content that is typically encountered in middle school English literature, social studies, history, and science texts. Both fiction and non-fiction text examples were provided in the manualized EF training program. The step-by-step approach of the EF training program used student friendly terms and simplified explanations and practices that built on one another.

#### SMART Educator Training

Teacher training was comprised of an intensive five-day workshop that combined information about brain development and neuroscience advancements with hands-on experience teaching the EF training program. The summer workshop provided teachers the opportunity to immerse themselves in the cognitive training.

During the first 2 ½ days, teachers participated in EF training as students, simulating the student’s experience, to establish a versatile comprehension of the metacognitive practices they would be teaching. The workshop was led by various members of the research team who were trained to work with teachers and had extensive experience teaching the EF training program to students.

During the remainder of the workshop, teachers were given the opportunity to review and “practice” EF training instruction and programmatic fundamentals in small group sessions guided by the research team members. Teachers were provided the program’s manualized instructional guidelines. Essential for understanding the necessity of time management in the classroom setting, small group practices afforded the educators an opportunity to consider questions and problems that might arise in their classrooms. Additionally, healthy brain practices derived from cognitive neuroscience research, such as the power of incorporating positive reinforcement and the importance of students’ verbalization of reasoning, were included.

Teachers’ scripts and instructions for implementation were included in a teaching manual to assist the educators’ ability to preserve fidelity and the intent of the program. The instructions emphasized student engagement, encouragement, and whole classroom discussion as paramount to the success of the program. Teachers were coached to encourage students to share their ideas in the classroom and verbalize their reasoning. Teachers were advised to expect and accept a variety of ideas and interpretations from their students. Thus, the teachers were directed to consider the importance of employing and modeling their own cognitive flexibility to buoy student confidence and creativity.

In addition to the program training protocols, teachers received short, 30-min informational sessions, regarding executive function, adolescent brain development, and other relevant cognitive neuroscience research. Upon conclusion of the formal EF educator training, the research team maintained regular communication with the trained teachers before and during classroom implementation. Additionally, the research team provided one to three in-person site-visits to reinforce and continue the teachers’ training during classroom implementation.

### Instruments

#### Immediate Metrics to Determine Program Efficacy

##### Scale of Advanced Reasoning

The Scale of Advanced Reasoning (SOAR©; Chapman et al., 2006; Gamino et al., 2010) was a 50-min pen and paper assessment administered to a classroom period of students as a group by a member of the research team. The SOAR requires participants to summarize three texts of differing lengths, two narrative and one expository. Production of abstracted deeper meanings during summarization reflects one’s ability to utilize gist-reasoning skills to understand and convey unstated, underlying meanings/ideas that may be implied but not directly stated within the information (Chapman et al., 2006; Gamino and Chapman, 2009; Gamino et al., 2010). Gist-reasoning required the student to construct inferences from the original text that were not directly stated. In other words, the student had to “read between the lines” in order to abstract and convey meanings within their summaries. Additionally, the SOAR requires production of interpretive statements or “lessons” that could be gleaned from the texts. Lastly, questions that required short answers regarding information recalled from the texts concluded each section of the assessment. Students recorded their answers on forms that were provided to them by the proctor.

Prior to administration of the SOAR, students received instructions regarding the qualities of a good summary as provided in the testing protocols. Specifically, the proctor informed the participants that a summary was a well-organized, shortened version of the original text that conveyed the bigger ideas and important information. Additionally, students were told that summaries should contain enough information so that a reader could understand what the original text was about without having to read the story themselves. The initial instructions included an example of a high-level, gist-based summary of a common fairy tale, “Little Red Riding Hood,” prior to presentation of the first assessment text.

The students were similarly instructed that they would be asked to write as many universal life lessons as possible that could be learned from the text. The proctor provided an example of a universal lesson for the sample summary of “Little Red Riding Hood.” The instructions concluded with a brief explanation of the questions that would be presented after the students had written their summary and lesson statements. Students were reminded that after the text was read, it would no longer be visible when they were writing their summaries, lesson statements, and answering the questions.

During testing, each passage was projected separately on a centrally located large screen for students to read and/or follow along while the proctor read the text aloud. After the first text was read, its image was removed from the screen and the proctor instructed the students to write a summary in their own words that included the bigger ideas and important information from the passage. The students were further instructed to write as many life lessons as a reader could learn from the story. After completing the summaries and lessons, the students were asked to write short answers to four recall questions about the story that were projected on the screen and included in their test booklet. After given time to complete these tasks, the process was repeated for the subsequent two passages.

Independent raters who were blinded to the school, student, classroom, and time of assessment administration (e.g., pre or post) scored the student summaries for the number of abstracted deeper meanings. Raters scored each gist-based idea according to a scoring rubric, coding for the presence of up to six gist-based ideas included in the summary for the first text, up to nine gist-based ideas included in the summary from the second text, and up to 10 ideas included in the summary for the third text, as each subsequent text increased in length and complexity. Ideas expressed explicitly in the original text were not considered a gist-based idea. The ratings were summed across the three summaries to create a total gist score for the assessment ranging from 0 to 25.

Each interpretive lesson statement was rated on a scale from 1 to 6 by applying a strict scoring rubric. Higher scores were indicative of an interpretive statement that incorporated meaning across the entire text and could be generalized to real-life contexts. An average score was obtained by dividing the sum of all lesson scores by the number of lessons written. However, due to classroom time constraints that limited the ability to complete administration of the final section, the fact recall segment of the assessment was not used in the analyses. Two trained raters independently coded each assessment. Inter-rater reliability for raters was calculated to be 92% for gist reasoning and 94% for interpretive lesson statements. Disagreements between the raters were subsequently resolved *via* consensus.

#### Metrics for Determination of Far Transfer

##### State of Texas Assessments of Academic Readiness

The State of Texas Assessments of Academic Readiness (STAAR; Texas Education Agency, 2019) was administered late in the spring semester of the school year. This state-mandated standardized test provides a uniform metric to inform administrative planning and student/teacher achievement as well as individual student grade-level advancement or retention. Different core content areas are tested for various grade levels. All eighth grade students in Texas took the STAAR core content assessments for Reading, Mathematics, Science, and Social Studies. Eighth grade students are required to pass the Reading and Mathematics assessments for promotion to ninth grade.

To pass the Reading test, the student had to demonstrate an ability to understand and analyze a variety of written texts across reading genres while making supportable inferences based on critical inquiry. For the Mathematics assessment, the student was required to show reasoning ability in applying mathematics to problems arising in everyday life, society, and the workplace. The Science assessment required a factual understanding of the science curriculum as well as an ability to employ investigation, reasoning, and planning skills to formulate reasonable explanations and predict trends. Similarly, the Social Studies assessment required a knowledge of specific issues and events from US history, an understanding of how various government and civic processes interact with geographic and cultural influences to affect historical events, and application of critical-thinking skills to organize and use information (Texas Education Agency, 2019).

For the current study, we utilized the performance classifications of the STAAR as a far-transfer outcome metric for two primary reasons. First, for students in the quasi-control group, the publicly published data obtained were limited to outcomes of performance classifications. Hence, related between-group comparisons were limited to pre-determined categorical results. Secondarily, performance levels are the most reliable and salient reflection of the state’s expectations. Year-to-year, STAAR tests do not contain equal numbers of questions or sections. Thus, the state relies on scaled scores, the conversion of which changes year-to-year, as well as from grade level-to-grade level, to determine the performance levels. As a result, performance levels are the primary metric used by the state. Performance levels remain constant from year-to-year and are used to determine the degree of student achievement. The STAAR tests have demonstrated acceptable reliability, with coefficients for the spring 2019 administrations ranging from 0.85 to 0.91 (Texas Education Agency, 2019). Since the data are publicly available, we obtained de-identified information regarding passing rates from the public database for the quasi-control group to compare with the school-provided data for the trained students.

### Procedures

#### SOAR Administration

Baseline SOAR administration took place during the fall semester, 1 day prior to the beginning of EF training implementation. After explaining the project and acquiring informed assent, a proctor from the research team administered the SOAR to each participating classroom period as a group, one class period at a time.

Post-training assessments were administered 3 weeks after the EF training program concluded. The post-test utilized the same group-administration procedure as at baseline. Students were monitored for compliance with testing instructions. Test samples in which the student did not follow administration guidelines and instructions were excluded from analyses.

#### STAAR Administration

The administration of the state-standardized STAAR test was highly regulated. Schools were required to test on specifically outlined days, and teachers were under strict guidelines for proctoring and ensuring that tests were valid indicators of students’ abilities. Completed scantron answer booklets and all testing materials were returned to the State of Texas where they were scored and passing rates determined. The data from the tests were dispersed throughout the state, first to the schools, then made publicly available online (Texas Analytic Portal, 2021).

### Analyses

Before analyzing the SOAR data, we excluded data from two class periods (*n* = 37) due to incomplete test administrations as a result of time constraints incurred during baseline testing. The delay in commencement of those class periods rendered insufficient time to take the full test. Additionally, SOAR data from five other participants were discarded due to student failure to follow proctor instructions. This yielded a final sample of 171 (88 females) students for paired comparisons of SOAR gist-reasoning scores, including an examination of teacher effects on executive function outcomes. Four students did not complete the entire interpretive statement production task, yielding a sample size of 167 for those analyses.

To explore the potential for far transfer of EF training effects, we obtained de-identified data from the school’s STAAR outcomes for students who received EF training. The data included the previous year’s performance level outcomes when the participants were in seventh grade for Mathematics and Reading (the only subjects tested during seventh grade), and the performance level outcomes from the spring semester after EF training for Mathematics, Reading, Science, and Social Studies. Additionally, we used the results of publicly available data for the untrained students (Texas Analytic Portal, 2021). For this analysis, we used Chi-square to compare the percentages of STAAR passing rates between the trained and untrained groups. The de-identified nature of the STAAR data precluded direct comparisons of STAAR outcomes for students who voluntarily completed the SOAR.

## Results

### Immediate Metric

To examine the immediate effects of EF training, a 2 × 3 × 2 mixed-model ANOVA was calculated to examine Time × Teacher × Gender effects on gist-reasoning and interpretive statement production. Our gist-reasoning analyses revealed a significant main effect of time. Specifically, students demonstrated higher gist-reasoning scores following EF training [*F*(1, 165) = 4.39, *p* = 0.038, *d* = 0.13; *M_post_* = 5.73, *SD_post_* = 2.77, *M_pre_* = 5.36, *SD_pre_* = 2.73]. A similar finding for interpretive statement production was found, reflecting a significant increase in scores after training [*F*(1, 161) = 9.16, *p* = 0.003, *d* = 0.24; *M_post_* = 3.82, *SD_post_* = 0.66, *M_pre_* = 3.67, *SD_pre_* = 0.60]. There were no significant effects of gender or teacher.

### Far Transfer (STAAR)

To determine if far transfer of EF training could be detected, we compared trained students’ passing rates with untrained students’ passing rates on the STAAR. Students were tested in the seventh grade for Reading and Mathematics proficiency and in the eighth grade for Reading, Mathematics, Science, and Social Studies proficiency.

**Figure 1:**
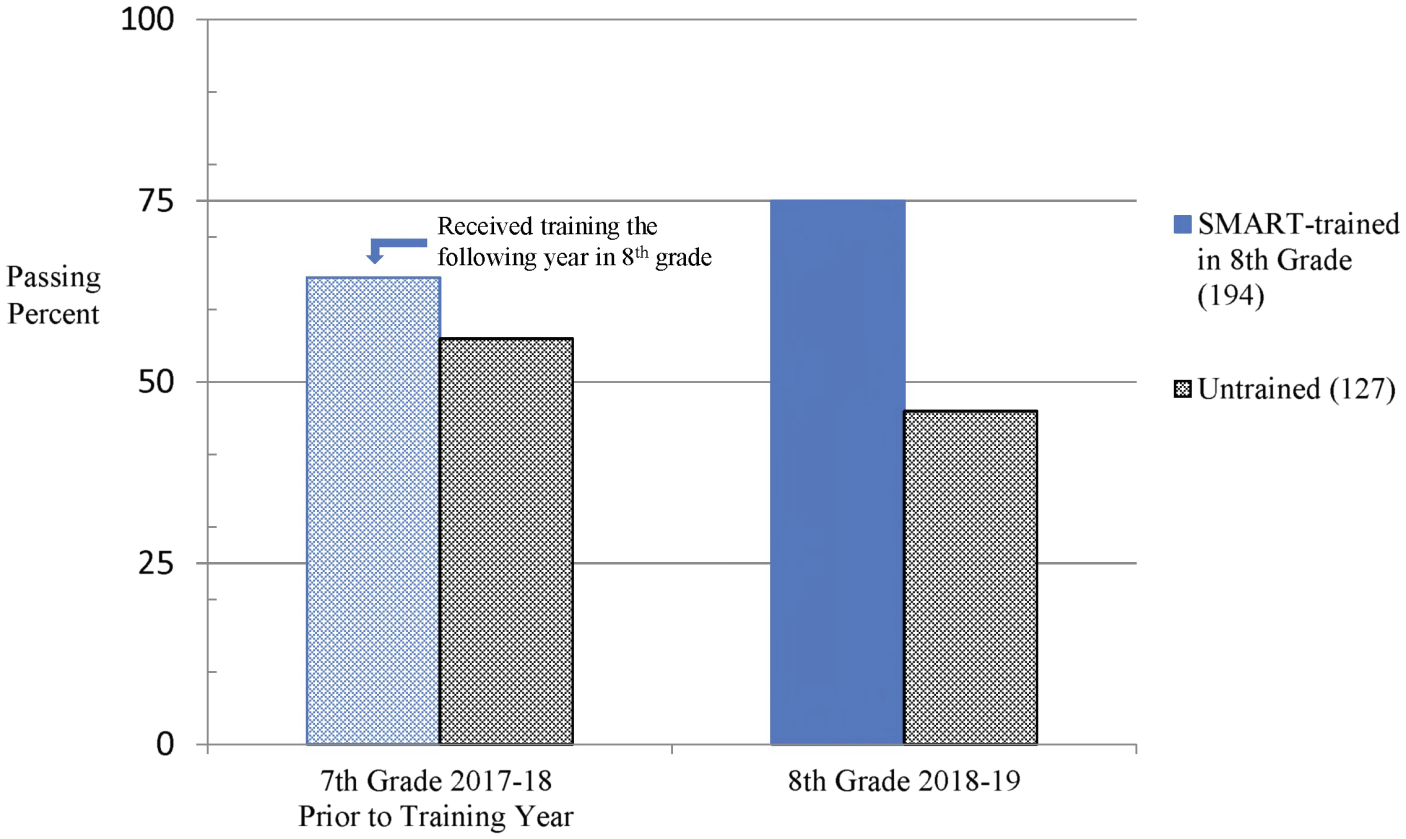
Passing rates are shown for trained and untrained groups on the State of Texas Assessment of Academic Readiness (STAAR) Reading test for seventh graders in the year before training and the next year in eighth grade 4 months after training. There is a significantly greater difference between the groups following training as more students from the trained group passed the reading test.

For analyses of standardized test outcomes, each teacher’s group of students was treated as a separate cohort. We performed separate Chi-square analysis to compare each cohort’s STAAR Reading passing rates to the control group. In the year prior to the EF training, we determined one trained cohort significantly outperformed the untrained control group in seventh grade STAAR Reading performance [χ^2^ (1, *N* = 243) = 10.67, *p* < 0.001, *w* = 0.21]. Due to the higher passing level of seventh grade achievement for this cohort, we excluded the data from additional comparisons of the STAAR Reading test to ensure the trained and untrained cohorts’ performance were comparable prior to the training. Chi-square tests confirmed no significant difference between the remaining trained cohorts and the untrained controls in STAAR Reading performance during seventh grade, [χ^2^ (1, *N* = 321) = 2.4, *p* = 0.125]. The eighth grade administration of the STAAR resulted in significantly higher passing rates for the trained, performance-matched group compared to the untrained control group in Reading, [χ^2^ (1, *N* = 321) = 28.59, *p* < 0.001, *w* = 0.30], as shown in Figure 1.

**Figure 2:**
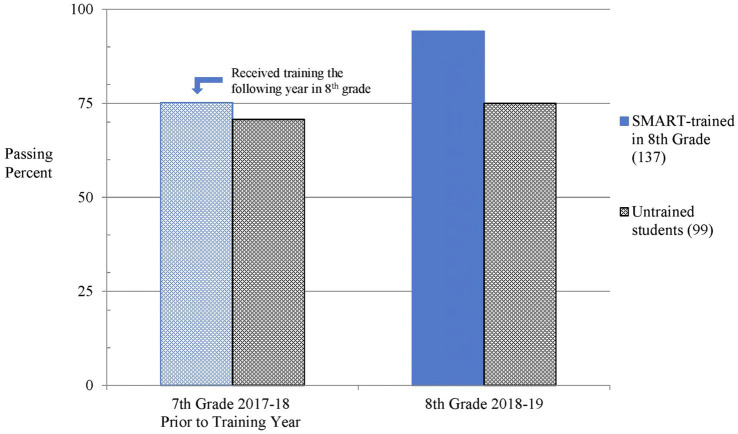
Passing rates are shown for trained and untrained groups on the STAAR Math test for seventh graders in the year before training and the next year in eighth grade 4 months after training. Similar to the reading outcomes, the difference between the groups is larger following training, with more students from the trained group passing the math test.

Similar to the STAAR Reading test, we performed separate Chi-square analysis to compare each cohort’s seventh grade STAAR Mathematics passing rates to the control group’s passing rates. In the year prior to the EF training, one trained cohort significantly outperformed the untrained control group in seventh grade, [χ^2^ (1, *N* = 222) = 5.42, *p* = 0.02, *w* = 0.16]. Due to the higher passing level of seventh grade Mathematics achievement for this cohort, we excluded the data from additional comparisons of the STAAR Mathematics test to ensure the trained and untrained cohorts’ performance were comparable prior to the training. Chi-square tests confirmed no significant differences between the remaining trained students and the untrained control students in STAAR Mathematics performance during seventh grade, [χ^2^ (1, *N* = 236) = 0.59, *p* = 0.443]. The results of the eighth grade administration of the STAAR Mathematics exam found the performance-matched trained group demonstrated a significantly higher passing rate than the control group [χ^2^ (1, *N* = 236) = 18.0, *p* < 0.001, *w* = 0.28], as shown in Figure 2.

The Science and Social Studies assessments were administered only in eighth grade; hence, only post-training passing rates could be compared between groups. Two of the cohorts significantly outperformed the untrained group, on the STAAR Science assessment [χ^2^ (1, *N* = 258) = 14.75, *p* < 0.001, *w* = 0.24], and [χ^2^ (1, *N* = 244) = 25.33, *p* < 0.001, *w* = 0.32]. The comparison of the third cohort’s passing rates was found to be insignificant from the untrained controls [χ^2^ (1, *N* = 197) = 2.29, *p* = 0.13, *w* = 0.11].

The STAAR Social Studies assessments demonstrated significantly higher passing rates for all three trained cohorts when compared to the untrained control group [χ^2^ (1, *N* = 197) = 5.99, *p* = 0.014, *w* = 0.17], [χ^2^ (1, *N* = 244) = 12.93, *p* < 0.001, *w* = 0.23], [χ^2^ (1, *N* = 258) = 16.0, *p* < 0.001, *w* = 0.25].

**Figure 3:**
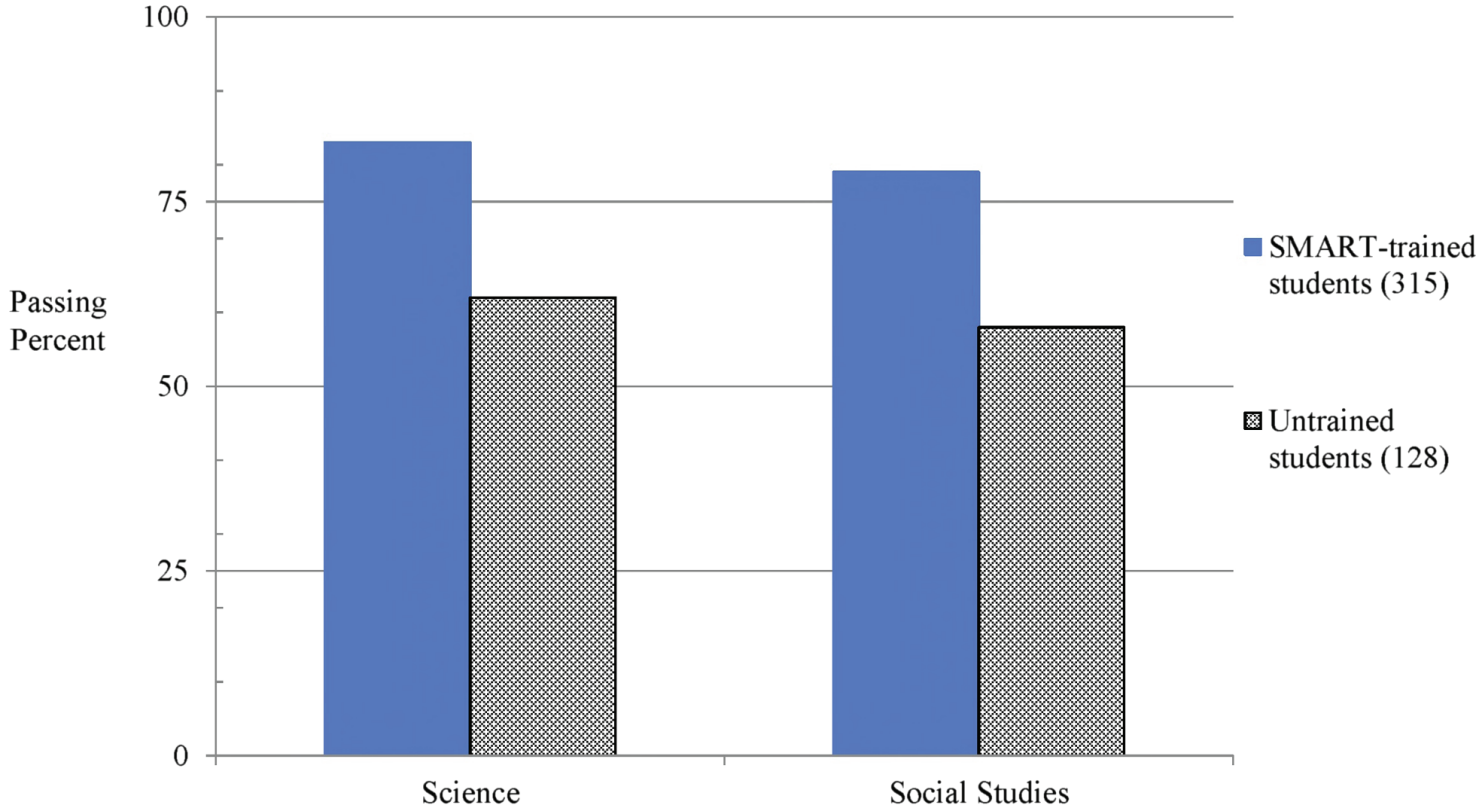
Passing rates for eighth graders are shown for trained and untrained groups on the STAAR Science and Social Studies tests after training. On both tests, the trained group outperformed the untrained group.

As illustrated in Figure 3, combining the cohorts resulted in a significant difference in passing rates, with the EF-trained group outperforming the untrained group in both Science [χ^2^ (1, *N* = 443) = 22.79, *p* < 0.001, *w* = 0.23] and Social Studies [χ^2^ (1, *N* = 443) = 20.78, *p* < 0.001, *w* = 0.22]. Figure 3 illustrates performance level data for Science and Social Studies at the end of the eighth grade school year.

In addition to examining differences between the trained and untrained groups on the STAAR, we examined the within-group longitudinal change using the Reading and Mathematics domains for the trained students. Using the raw scores, a paired t-test revealed a significant increase in the Reading outcomes from seventh to eighth grade, [*t*(309) = 15.42, *p* < 0.001, *d* = 0.54; *M_post_* = 31.23, *SD_post_* = 7.4, *M_pre_* = 26.93, *SD_pre_* = 8.4]. Similarly, a paired *t*-test revealed a significant increase in the Mathematic outcomes from seventh to eighth grade, [*t*(259) = 18.51, *p* < 0.001, *d* = 0.94; *M_post_* = 28.25, *SD_post_* = 6.42, *M_pre_* = 21.75, *SD_pre_* = 7.39].

The magnitude of change in Reading and Mathematics raw scores from seventh grade to eighth grade for the trained students in this study was, 4.3 in Reading and 6.5 in Mathematics. The trained students’ improvements exceeded the statewide average improvement of 3.5 in Reading and 5.5 in Mathematics for the same school year. Thus, the EF trained students showed improvement in STAAR performance not only compared to their same-school peers but compared to average change scores in eighth grade students throughout the State of Texas.

## Discussion

We had two primary aims for this study. First, we wanted to determine if teacher-guided EF training would demonstrate immediate gains in higher-order cognition. Second, we wanted to determine if teacher-guided EF training would demonstrate far transfer as evidenced by improved performance on state-mandated standardized tests. We found that training teachers to instill higher-order executive function in students improved student performance on near and far measures of cognition and academic performance.

We trained teachers to think about student learning from a cognitive perspective, rather than a content perspective. Thus, during program implementation, the teachers were effectively providing instruction for how to learn, rather than solely presenting students with key content to memorize. Teachers were trained to adhere to the manualized EF training program and instruct basic executive function to students first to support selective focus, organization, paraphrasing, and synthesis before moving on to instructing higher-level executive function. Higher-level executive functions included connecting new information with world knowledge in order to abstract meanings, interpret, and analyze. Analyzing and interpreting information are standard objectives for most educators; however, helping students obtain these cognitive goals is a complicated task that often leaves teachers struggling to try to help all students attain this level of cognition. Our EF training presented cognitive practices founded on neuroscientific evidence for both teachers and students in a simplified, stepwise manner that provided an understanding of how higher-level processing is attained. Our previous investigations found our metacognitive approach to cognitive training through the EF training protocols, guided students to reflect on their thinking with practical exercises to reinforce depth of processing, improved gist-reasoning and fact learning.

The current findings add to prior evidence that our EF training provided an effective way to increase students’ comprehension of higher-order executive function leading to improved learning and application of skills across content areas (Gamino et al., 2010, 2014). The results revealed that eighth grade students who received EF training through the teacher-guided SMART program showed significant improvement relative to baseline in gist-reasoning and interpretive statement production 3 weeks after the intervention. These results support our previous research (Gamino et al., 2010, 2014) that found students who received cognitive training in their classrooms from our research team demonstrated improved gist reasoning. Furthermore, the results support our hypothesis that teachers could proficiently implement EF training in their classrooms to yield similar efficacy.

The cohort of students in this study not only showed improved high-level executive function skills (e.g., gist reasoning and interpretive statements) immediately after their training, but also improved standardized testing results in Reading and Mathematics compared to their pre-training scores from seventh grade. The findings of improved academic performance for the participants months after the original EF training suggest far transfer of the skills obtained from cognitive instruction. The analyses corroborate our previous research (Gamino et al., 2014) reflecting significant cognitive changes for children who were trained to think about and expand their understanding of new information.

This study provides evidence that universally presented interventions focusing on cognitive enhancement can be successfully implemented by existing teachers after training. The teachers in this study were trained to adhere to protocols and received hands-on experience and application of the materials, with periodic coaching and support in their classrooms. Evidence-based universal interventions, unlike those targeted at select groups of students (e.g., those who are “gifted” or those who are struggling), can raise performance levels for all students regardless of their initial level.

Our universal approach to implementation ensured that all students in the classroom received EF training. Students who were performing at higher levels prior to the training potentially benefited by acquiring awareness and knowledge about cognition that they did not previously possess. The students who were performing at lower academic levels prior to training received knowledge, direction, and practice to support and expand their ability to think deeply, interpret, and analyze. These higher-order executive functions are imperative for academic performance and social skills that benefit future endeavors, underscoring the vital importance of infusing executive function in education (Diamond, 2012). Our results suggest that providing teachers with the means to approach teaching with a cognitive neuroscience-based understanding promotes the potential to increase student performance in the near and far term.

The teachers who were trained to implement the EF program, provided anecdotal feedback attesting to their students’ increased self-confidence, greater willingness to participate in class discussions, and improved engagement during and after implementation. They further reported that students seemed to derive a sense of agency for their learning and a newfound curiosity about the regular classroom content. We plan to validate these perspectives in future work. The interactions ostensibly led to the teachers having a better understanding of their students’ perspectives, in addition to demonstrating openness to different opinions and respect for students’ individuality (Yeager et al., 2018). A new administrator who joined the school after the EF training concluded recognized and reported to the research team that the teaching styles of the trained teachers had qualities that other teachers in the school did not possess. Although not directly measured, we infer from this anecdotal information that the cognitive training produced the underpinnings of a classroom culture that, from both the educators’ and the students’ perspective, aligned to foster deeper thinking and cognitive flexibility.

Our EF training program was developed to ingrain the brain processes that allow higher-order thinking abilities. The rigorous training provided to the teachers was developed to instill the importance of student engagement through open-ended questions, acceptance of multiple correct answers, and discussion regarding the reason behind the choices students made. Teachers were provided with specific written guidelines and prompts to direct teacher and student thought processes toward improving comprehension of unstated meanings rather than focus on specific details.

We used the Scale of Advanced Reasoning to measure immediate cognitive changes in students after receiving the EF training and found significant growth. Our previous evidence showed there were no significant practice effects with the SOAR (Gamino et al., 2014). Indeed, the assessment served as a direct metric to determine the immediate efficacy of the EF training program for the students and the trained teachers’ compliance with protocols. The two SOAR domains we assessed for evidence of higher-order executive function abilities were gist reasoning and interpretive statement production. Gist reasoning requires one to make inferences and derive abstracted meanings from the text (Chapman et al., 2006; Chapman and Mudar, 2013; Gamino et al., 2014). Production of abstracted deeper meanings during summarization reflects one’s ability to utilize gist reasoning skills to understand and convey unstated, underlying meanings/ideas that may be implied but not directly stated in the information (Chapman et al., 2006; Chapman and Mudar, 2013). As such, gist reasoning indicates a students’ inclination to think about and process information at a deeper level rather than memorize and directly recall verbatim details. Depth of thinking requires connecting previous knowledge with new information to draw inferences and conclusions about information (Kintsch and van Dijk, 1978).

The present results indicated that the students in this study significantly increased their production of gist-based information when summarizing the texts. That is, they inserted more inferential meanings abstracted from the texts into their summaries. During EF training, students were guided through the practice of abstracting meanings through teacher-led questioning. The teachers followed the outlined questions in their teaching manuals, such that the students were scaffolded to practice abstracting meanings. Student production of abstracted meanings during the SOAR required independent and spontaneous investigation of deeper implied meanings from the texts. Thus, students’ participation in the guided exercises for abstracting meanings during EF training translated into metacognitive application during the SOAR.

The SOAR interpretive statement component measured the ability to produce lesson statements that contained meanings and perspectives generalized to life that could be gleaned from the text, an indicator of cognitive flexibility. Cognitive flexibility in general helps one speculate about others’ experiences, opinions, and potential motives and behaviors. Cognitive flexibility facilitates openness to changing course when an original plan of action is no longer viable. Cognitive flexibility is an important aspect of healthy functioning in society, and the backbone of persistence (Nijstad et al., 2010; Steinbeis and Crone, 2016). Students in this study significantly increased the quality of their interpretive statement production, suggesting an increase in participants’ cognitive flexibility.

During our EF training, interpretative statement production was fostered by guiding students to think about and create a variety of assumptions regarding lessons that could be extrapolated from different aspects of a story, including characters, turns of events, or various actions. Guided by the teachers’ scaffolding questions, students made personal and global connections with various passages, deriving multiple interpretations. Students’ varied interpretations were shared openly with the class, to foster collaborative thought generation and innovation. The impetus for these exercises was to induce cognitive flexibility, *via* critical thinking and acceptance of a variety of perspectives and opinions (Yeager et al., 2018). The results of the SOAR interpretive statement section suggest that the EF training improved students’ ability to think flexibly and globally about text-based information.

To substantiate the evidence of increased academic performance from our previous studies (Gamino et al., 2010, 2014), our current study aimed to determine if evidence of far transfer occurred by analyzing the results of state mandated standardized test outcomes. The STAAR is prioritized in most Texas public schools as the primary metric to determine the attainment of student and teacher success. The STAAR Reading and Mathematics tests are administered annually to students in third through eighth grade, while other content areas are tested at various grade-level intervals.

We compared seventh grade outcomes from each teacher’s cohort of trained students separately to ensure the post-training results were not biased due to higher achievement prior to training. We removed one cohort’s Reading data set due to significantly higher baseline (seventh grade) performance for the Reading assessment. We removed a different cohort’s Mathematics data set due to significantly higher baseline (seventh grade) performance for the Mathematics assessments. Thus, initial seventh-grade performance levels were comparable between the trained and untrained groups for each analysis of the Reading and Mathematics assessments. The trained students in this study demonstrated improved passing levels on the Reading and Mathematics STAAR examinations that significantly exceeded the performance levels of their untrained peers. In addition, two trained cohorts surpassed the performance levels of their untrained peers on the Science test, and all three cohorts surpassed the performance levels of their untrained peers on the Social Studies test. Thus, across all four tested content areas the trained participants outperformed their untrained peers. Significant longitudinal growth was determined within the group of trained students for Reading and Mathematics performance from seventh to eighth grade that exceeded the statewide average. These findings suggest that far transfer of skills gleaned from EF training may manifest in improved academic performance for the majority of students.

We acknowledge that standardized testing formats are typically more content driven rather than a thorough metric of high-level executive function. However, embedded within the format of the STAAR exists an intent to ensure students have a deeper understanding of the content. A percentage of the STAAR questions aim to determine students’ ability to think, plan, and analyze beyond the stated facts. The need to apply critical-thinking and reasoning to some of the STAAR questions across each subject suggests the need for higher-order executive function abilities.

Our teacher training program emphasized educator support and helping the teachers attain their goals of increasing their students’ abilities and academic performance. Teachers were trained and coached to enhance student appreciation of global thinking. Student engagement was paramount to the success of the program. Adhering to our training protocols, the trained teachers asked their students conceptual questions and provided opportunities for students to voice opinions and demonstrate diverse thinking. Furthering the success of the program, students directly observed and interacted with their teachers’ openness to a variety of ideas and interpretations. Coaching sessions occurred during the teachers’ initial training as well as during implementation of the program through in-person classroom visits. The site visits helped ensure teachers understood and followed protocols and students were progressing through the program. Although initially hesitant to allocate 12 days (10 training sessions plus 2 testing days) out of their schedules for assessment and implementation of EF training, the teachers’ consensus after completion was that the investment of time was miniscule compared to the gains for their students.

## Limitations

One of the primary limitations of this study is the lack of a randomized control group. Ideally, students from each class period would have been randomized into either the experimental group or the control group. The students in the quasi-control group were automatically excluded from EF training due to their predetermined placement into the fourth ELA teacher’s classroom. We were unable to administer the SOAR to control students due to their teacher’s decision not to participate; thus, we have no information regarding their higher-order executive function skills of gist-reasoning or interpretation. Likewise, we do not know if there were other factors in the lives/academic experiences of the quasi-control group that would affect their lesser performance on the STAAR test.

An additional limitation of this research study is the necessity for group administration of the SOAR, which did not allow adjustments for individual students who might have preferred additional time to produce a summary and interpretive lessons. Assessments had to be fully administered during one classroom period; thus, if students were tardy, needed restroom breaks, or were absent, accommodations could not be made to ensure each student who consented to participate would be able to complete the full administration of the SOAR.

We also acknowledge the limitation of using standardized test scores, which provide a snapshot of a student’s abilities but are not sufficiently sensitive to capture a precise measurement of depth of understanding that reflects higher-order executive function. To protect the students’ right to privacy, the school submitted deidentified standardized test scores; thus, we were unable to determine if a direct relationship between performance on the SOAR and performance on the STAAR was evident. The de-identified STAAR data also hampered our ability to determine within group differences between the students who agreed to participate in the cognitive testing and those who declined. Future studies will address these limitations through directly consenting parents to enable full access to all standardized test data. Likewise, the students who participated in this study could have had other unknown factors, such as tutoring or coaching in their lives that contributed to their improved gist-reasoning and STAAR scores.

In addition, this study was limited by the lack of sociodemographic information obtained for each student. As this was a group study that relied on the adoption of our EF training curriculum by the school and the trained teachers, we did not have the consent from the participants’ parents to facilitate the collection of data that would determine the relationship of diagnosed neurodevelopmental disorders, IQ, or more specific family income levels to our cognitive training. Such data collection will be considered for future studies to determine the efficacy of the EF training for a variety of student abilities.

Additional longitudinal data would be valuable to determine far transfer beyond the 4 months after EF training when students took the STAAR test. Future studies must explore far transfer and longitudinal effects of EF training on academic success, as the implications for college and career readiness are critical to the future of the students.

The teachers in this study were recruited to act as facilitators of the research, not participants; hence, no empirical measures were collected to directly determine the teachers’ level of understanding or if the training and program implementation produced changes in their personal cognition. However, based on their anecdotal, subjective feedback, it is likely that the teachers benefitted personally and professionally from learning more about their own and their students’ cognition in addition to having a better understanding of brain science. Future studies will need to address teacher outcomes for a more thorough understanding of significant educator changes that take place after training and implementation.

Additional limitations are the potential for “self-selection effects” of the teachers who voluntarily chose to participate. The voluntary aspect regarding teacher participation could be an indicator of the participating teachers’ innate traits of flexibility and openness rather than a direct result of the professional development. At the same time, such characteristics could have enhanced the benefits that the participating teachers received from the professional development. An additional consideration that was not explored in this study is the innate qualities of teaching proficiency for the three participating teachers, compared to the teaching style of the non-participatory teacher. The quality of pre-existing teaching expertise could have contributed to or been the direct cause of the higher performance of participating students.

To date, only the teachers who attended our workshop to learn how to implement the EF training had access to the program. Consequently, we have no data regarding the implications of teachers who implement our program without our training. In the future, gathering evidence to compare teachers who implement the program after attending our workshop to teachers who implement the program without attending our training workshop may provide valuable information regarding the efficacy and/or necessity of our intensive workshop.

## Conclusion and Future Directions

In the current research, teachers’ influence, personal relationships, and familiarity with their students potentially increased the efficacy of the EF training beyond the results that the research team had achieved previously. We strongly believe the ecological validity achieved by performing research in the classroom universally is important and outweighs most of the drawbacks. The implications of this study for educators, students, and clinicians suggest the inherent value of empowering young adolescents with high-level executive function skills that provide long-term benefits regardless of students’ pre-existing abilities. Providing EF training to classrooms universally has the potential to elucidate cognitive processing and neuroscience principles for students who are inherently strong learners as well as strengthen the abilities of students with weaker learning skills.

This study adds to our knowledge regarding the importance of executive function development and improvement for the successful acquisition of life-long skills that potentially influence the ability to become a productive member of society. We remain steadfast in our belief that teachers can and should explicitly present evidence-based high-level executive training to all students.

## Data Availability Statement

The raw data supporting the conclusions of this article will be made available by the authors, without undue reservation.

## Ethics Statement

The studies involving human participants were reviewed and approved by the University of Texas at Dallas, Human Subjects Research Office of Research Integrity and Outreach. Written informed consent from the participants’ legal guardian/next of kin was not required to participate in this study in accordance with the national legislation and the institutional requirements.

## Author Contributions

JG, CF, and RR contributed equally to the writing of the manuscript. JK contributed to an earlier version of the manuscript. SC contributed to the final edits and revisions of the manuscript. RR performed the analysis under the direction of JG. JG is the primary investigator of this research. All authors contributed to the article and approved the submitted version.

## Funding

Private Grants were awarded by the Hoglund Foundation, Capital for Kids, and the Communities Foundation of Texas. Public Funding was awarded by the State of Texas.

## Conflict of Interest

The authors declare that the research was conducted in the absence of any commercial or financial relationships that could be construed as a potential conflict of interest.

## Publisher’s Note

All claims expressed in this article are solely those of the authors and do not necessarily represent those of their affiliated organizations, or those of the publisher, the editors and the reviewers. Any product that may be evaluated in this article, or claim that may be made by its manufacturer, is not guaranteed or endorsed by the publisher.

## References

[ref1] Best J. Miller P. (2010). A developmental perspective on executive function. Child Dev. 81, 1641–1660. doi: 10.1111/j.1467-8624.2010.01499.x, PMID: 21077853 PMC3058827

[ref2] Blackwell L. S. Trzesniewski K. H. Dweck C. S. (2007). Implicit theories of intelligence predict achievement across an adolescent transition: a longitudinal study and an intervention. Child Dev. 78, 246–263. doi: 10.1111/j.1467-8624.2007.00995.x, PMID: 17328703

[ref3] Brick K. Cooper J. L. Mason L. Faeflen S. Monmia J. Dubinsky J. M. (2021a). Tiered neuroscience and mental health professional development in Liberia improves teacher self-efficacy, self-responsibility, and motivation. Front. Hum. Neurosci. 15:664730. doi: 10.3389/fnhum.2021.664730, PMID: 34045949 PMC8144652

[ref4] Brick K. Cooper J. L. Mason L. Faeflen S. Monmia J. Dubinsky J. M. (2021b). Training-of-trainers neuroscience and mental health teacher education in Liberia. Front. Hum. Neurosci. 15:653069. doi: 10.3389/fnhum.2021.653069, PMID: 34220469 PMC8249721

[ref5] Buttelmann F. Karbach J. (2017). Development and plasticity of cognitive flexibility in early and middle childhood. Front. Psychol. 8:1040. doi: 10.3389/fpsyg.2017.01040, PMID: 28676784 PMC5476931

[ref6] Chang Z. Schwartz M. Hinesley V. Dubinsky J. (2021). Neuroscience concepts changed teachers’ views of pedagogy and students. Front. Psychol. 12:685856. doi: 10.3389/fpsyg.2021.685856, PMID: 34456800 PMC8384951

[ref7] Chapman S. B. Gamino J. F. (2008). Strategic Memory Advanced Reasoning Training. The University of Texas at Dallas, Center for BrainHealth.

[ref8] Chapman S. Gamino J. Cook L. Hanten G. Levin H. (2006). Impaired discourse gist and working memory in children after brain injury. Brain Lang. 97, 178–188. doi: 10.1016/j.bandl.2005.10.002, PMID: 16288805

[ref9] Chapman S. Gamino J. Mudar R. (2012). “Higher order strategic gist reasoning in adolescence,” in The Adolescent Brain: Learning, Reasoning, and Decision Making. eds. Reyna V. F. Chapman S. B. Dougherty M. R. Confrey J. (American Psychological Association), 123–151.

[ref10] Chapman S. Mudar R. (2013). Discourse gist: a window into the brain’s complex cognitive capacity. Discourse Stud. 15, 519–533. doi: 10.1177/1461445613501444

[ref11] Chapman S. Mudar R. (2014). Enhancement of cognitive and neural functions through complex reasoning training: evidence from normal and clinical populations. Front. Syst. Neurosci. 8:69. doi: 10.3389/fnsys.2014.00069, PMID: 24808834 PMC4009420

[ref12] Claro S. David Paunesku D. Carol S. Dweck C. S. (2016). Mindset tempers effects of poverty on achievement. Proc. Natl. Acad. Sci. 113, 8664–8668. doi: 10.1073/pnas.1608207113, PMID: 27432947 PMC4978255

[ref13] Darling-Hammond L. Hyler M. E. Gardner M. (2017). Effective Teacher Professional Development. Learning Policy Institute. Available at: https://learningpolicyinstitute.org/sites/default/files/product-files/Effective_Teacher_Professional_Development_REPORT.pdf (Accessed January, 2022).

[ref14] Dekker S. Lee N. Howard-Jones P. Jolles J. (2012). Neuromyths in education: prevalence and predictors of misconceptions among teachers. Front. Psychol. 3:429. doi: 10.3389/fpsyg.2012.00429, PMID: 23087664 PMC3475349

[ref15] Diamond A. (2006). “The early development of executive functions,” in Lifespan Cognition: Mechanisms of Change. eds. Bialystok E. Crik F. I. M. (Oxford University Press), 70–95.

[ref16] Diamond A. (2012). Activities and programs That improve Children’s executive functions. Curr. Dir. Psychol. Sci. 21, 335–341. doi: 10.1177/0963721412453722, PMID: 25328287 PMC4200392

[ref17] Diamond A. Lee K. (2011). Interventions shown to aid executive function development in children 4 to 12 years old. Science 333, 959–964. doi: 10.1126/science.1204529, PMID: 21852486 PMC3159917

[ref18] Diamond A. Ling D. S. (2016). Conclusions about interventions, programs, and approaches for improving executive functions that appear justified and those that, despite much hype, do not. Dev. Cogn. Neurosci. 18, 34–48. doi: 10.1016/j.dcn.2015.11.005, PMID: 26749076 PMC5108631

[ref19] Diamond A. Ling D. S. (2020). “Review of the evidence on, and fundamental questions about, efforts to improve executive functions, including working memory,” in Cognitive and Working Memory Training: Perspectives From Psychology, Neuroscience, and Human Development. eds. Novick J. M. Bunting M. F. Dougherty M. R. Engle R. W. (Oxford: Oxford University Press), 143–431.

[ref20] Dubinsky J. M. Roehrig G. Varma S. (2013). Infusing neuroscience into teacher professional development. Educ. Res. 42, 317–329. doi: 10.3102/0013189X13499403, PMID: 26139861 PMC4485447

[ref21] Gamino J. F. Chapman S. B. (2009). Reasoning in children with attention deficit hyperactivity disorder. Adv.ADHD 3, 82–88.

[ref22] Gamino J. F. Chapman S. B. Hull E. L. Lyon G. R. (2010). Effects of higher-order cognitive strategy training on gist reasoning and fact learning in adolescents. Front. Psychol. 1:188. doi: 10.3389/fpsyg.2010.00188, PMID: 21833248 PMC3153797

[ref23] Gamino J. F. Motes M. M. Riddle R. Lyon G. R. Spence J. S. Chapman S. B. (2014). Enhancing inferential learning in adolescence: new hope for students in poverty. Front. Hum. Neurosci. 8:924. doi: 10.3389/fnhum.2014.00924, PMID: 25505393 PMC4243561

[ref24] Garet M. S. Porter A. C. Desimone L. Birman B. F. Yoon K. S. (2001). What makes professional development effective? Results from a national sample of teachers. Am. Educ. Res. J. 38, 915–945. doi: 10.3102/00028312038004915

[ref25] Guerriero S. (ed.) (2017). Pedagogical Knowledge and the Changing Nature of the Teaching Profession. Paris: OECD Publishing

[ref26] Hackman D. A. Farah M. J. (2009). Socioeconomic status and the developing brain. Trends Cogn. Sci. 13, 65–73. doi: 10.1016/j.tics.2008.11.003, PMID: 19135405 PMC3575682

[ref27] Hackman D. A. Farah M. J. Meaney M. J. (2010). Socioeconomic status and the brain: mechanistic insights from human and animal research. Nat. Rev. Neurosci. 11, 651–659. doi: 10.1038/nrn2897, PMID: 20725096 PMC2950073

[ref28] Hackman D. Gallop R. Evans G. Farah M. (2015). Socioeconomic status and executive function: developmental trajectories and mediation. Dev. Sci. 18, 686–702. doi: 10.1111/desc.12246, PMID: 25659838

[ref29] Imal A. Wexler B. (2018). Increasing readiness to learn: benefits of executive function training in kindergarten carry over to first grade. Creat. Educ. 9, 2662–2676. doi: 10.4236/ce.2018.916201

[ref30] Jacob R. Parkinson J. (2015). The potential for school-based interventions that target executive function to improve academic achievement: a review. Rev. Educ. Res. 85, 512–552. doi: 10.3102/0034654314561338

[ref31] Jensen B. Jensen P. Rasmussen A. (2017). Does professional development of preschool teachers improve children’s socio-emotional outcomes? Lab. Econom. 45, 26–39. doi: 10.1016/j.labeco.2016.11.004

[ref32] Karbach J. Unger K. (2014). Executive control training from middle childhood to adolescence. Front. Psychol. 5:390. doi: 10.3389/fpsyg.2014.00390, PMID: 24847294 PMC4019883

[ref33] Kavanaugh B. Tuncer O. Wexler B. (2019). Measuring and improving executive functioning in the classroom. J. Cognit. Enhanc. 3, 271–280. doi: 10.1007/s41465-018-0095-y

[ref34] Kintsch W. van Dijk T. A. (1978). Toward a model of text comprehension and production. Psychol. Rev. 85, 363–394. doi: 10.1037/0033-295X.85.5.363

[ref35] Luby J. Belden A. Botteron K. Marrus N. Harms M. P. Babb C.. (2013). The effects of poverty on childhood brain development: the mediating effect of caregiving and stressful life events. JAMA Pediatr. 167, 1135–1142. doi: 10.1001/jamapediatrics.2013.3139, PMID: 24165922 PMC4001721

[ref36] MacNabb C. Schmitt L. Michlin M. Harris I. Thomas L. Chittendon D.. (2006). Neuroscience in middle schools: a professional development and resource program that models inquiry-based strategies and engages teachers in classroom implementation. CBE—life sciences. Education 5, 144–157. doi: 10.1187/cbe.05-08-0109, PMID: 17012205 PMC1618517

[ref37] Mayer R. E. (1989). Models for understanding. Rev. Educ. Res. 59, 43–64. doi: 10.3102/00346543059001043, PMID: 35421904

[ref38] Meltzoff A. N. Kuhl P. K. Movellan J. Sejnowski T. J. (2009). Foundations for a new science of learning. Science 325, 284–288. doi: 10.1126/science.1175626, PMID: 19608908 PMC2776823

[ref39] Miyake A. Friedman N. P. Emerson M. J. Witzki A. H. Howerter A. Wager T. D. (2000). The unity and diversity of executive functions and their contributions to complex “frontal lobe” tasks: a latent variable analysis. Cogn. Psychol. 41, 49–100. doi: 10.1006/cogp.1999.0734, PMID: 10945922

[ref40] Moffitt T. E. Arseneault L. Belsky D. Dickson N. Hancox R. J. Harrington H.. (2011). A gradient of childhood self-control predicts health, wealth, and public safety. Proc. Natl. Acad. Sci. U. S. A. 108, 2693–2698. doi: 10.1073/pnas.1010076108, PMID: 21262822 PMC3041102

[ref41] Morgan-Borkowsky L. (2012). “Executive Functions in the Schools: What Do Teachers Know About Executive Functions and How They Impact Student Progress?”. PCOM Psychology Dissertations. Paper 230. Available at: https://digitalcommons.pcom.edu/cgi/viewcontent.cgi?article=1229&context=psychology_dissertations (Accessed January, 2022).

[ref42] Musset P. (2010). Initial Teacher Education and Continuing Training Policies in a Comparative Perspective: Current Practices in OECD Countries and a Literature Review on Potential Effects. OECD, Directorate for Education. OECD Education Working Papers.

[ref43] National Center of Educational Statistics (2019). The Condition of Education. Federal Schools and Staffing Surveys from 1999–2000 and 2003–04, 34. Available at: https://nces.ed.gov/pubs2019/2019144.pdf (Accessed January, 2022).

[ref44] National Staff Development Council (2019). National Standards Assessment Inventory. Oxford, OH: NSDC.

[ref45] Nijstad N. DeDreu C. Rietzschel E. Baas M. (2010). The dual pathway to creativity model: creative ideation as a function of flexibility and persistence. Eur. Rev. Soc. Psychol. 21, 34–77. doi: 10.1080/10463281003765323

[ref46] OECD (1998). Staying Ahead: In-Service Training and Teacher Professional Development. Paris: OECD Publishing

[ref47] Organisation for Economic Co-operation, and Development (2002). Understanding the Brain: Toward New Learning Science. Paris: OECD Publishing.

[ref48] Organisation for Economic Cooperation and Development (OECD) (2005). Teachers Matter: Attracting, Developing, and Retaining Effective Teachers. Paris: OECD Publishing.

[ref49] Privitera A. J. (2021). A scoping review of research on neuroscience training for teachers. Trend. Neurosci. Educ. 24:100157. doi: 10.1016/j.tine.2021.100157, PMID: 34412863

[ref50] Roehrig G. H. Michlin M. Schmitt L. MacNabb C. Dubinsky J. M. (2012). Teaching neuroscience to science teachers: facilitating the translation of inquiry-based teaching instruction to the classroom. CBE—life sciences. Education 11, 413–424. doi: 10.1187/cbe.12-04-0045, PMID: 23222837 PMC3516797

[ref51] Smid C. R. Karbach J. Steinbeis N. (2020). Toward a science of effective cognitive training. Curr. Dir. Psychol. Sci. 29, 531–537. doi: 10.1177/0963721420951599

[ref52] Steinbeis N. Crone E. (2016). The link between cognitive control and decision-making across child and adolescent development. Curr. Opin. Behav. Sci. 10, 28–32. doi: 10.1016/j.cobeha.2016.04.009, PMID: 25632132

[ref53] Texas Analytic Portal (2021). Data Interaction for Texas Student Assessments. Available at: https://txreports.emetric.net (Accessed December 1, 2021).

[ref54] Texas Education Agency (2019). Technical Digest 2018–2019. Available at: https://tea.texas.gov/student-assessment/testing/student-assessment-overview/technical-digest-2018-2019 (Accessed January 20, 2022).

[ref55] Tham R. Walker Z. Tan S. H. D. Low L. T. Annabel Chen S.-H. (2019). Translating education neuroscience for teachers. Learn. Res. Pract. 5, 149–173. doi: 10.1080/23735082.2019.1674909

[ref56] Titz C. Karbach J. (2014). Working memory and executive functions: effects of training on academic achievement. Psychol. Res. 78, 852–868. doi: 10.1007/s00426-013-0537-1, PMID: 24389706

[ref57] Voss T. Kunter M. Baumert J. (2011). Assessing teacher candidates’ general pedagogical/psychological knowledge: test construction and validation. J. Educ. Psychol. 103, 952–969. doi: 10.1037/a0025125

[ref58] Wei R. C. Darling-Hammond L. Andree A. Richardson N. Orphanos S. (2009). Professional learning in the learning profession: A status report on teacher development in the United States and abroad. National Staff Development Council. Available at: https://edpolicy.stanford.edu/sites/default/files/publications/professional-learning-learning-profession-status-report-teacher-development-us-and-abroad.pdf (Accessed January, 2022).

[ref59] Wexler B. Iseli M. Leon S. (2016). Cognitive priming and cognitive training immediate and far transfer to academic skills in children. Nat. Sci. Rep. 6:32859. doi: 10.1038/srep32859, PMID: 27615029 PMC5018694

[ref60] Yeager D. Dahl R. Dweck C. (2018). Why interventions to influence adolescent behavior often fail but could succeed. Perspect. Psychol. Sci. 13, 101–122. doi: 10.1177/1745691617722620, PMID: 29232535 PMC5758430

