# Supplementary material for: Higher-Order Executive Function in Middle School: Training Teachers to Enhance Cognition in Young Adolescents
Source: Front Psychol. 2022 May 3;13:867264. doi: 10.3389/fpsyg.2022.867264 (PMC9111740; doi:10.3389/fpsyg.2022.867264)
Supplement: Supplementary file 1 [file Table_1.DOCX]

Addendum

EF Training Curriculum Overview

The Strategic Memory Advanced Reasoning Training (SMART) program consists of ten 45-minute sessions presented over a four-week period. The cognitive processes are taught in a hierarchical fashion with lower-level executive function processes reviewed first. The following is a brief description of each process.

| **Session** | **Processes** | **Description** | **Instruction** |
| --- | --- | --- | --- |
| 1 | Inhibit and Select | Focusing attention toward important information while inhibiting repeated information and superfluous details.  Reasoning abilities are enhanced through verbalizing explanations for selected choices. | Read a text to completion, delete repeated, extra details, and information unrelated to the topic.  Text examples used in the curriculum are written specifically for the purpose of reinforcing the inhibit and select processes. The brain basis of selective attention and alleviating cognitive overload is incorporated into instruction. |
| **2** | Chunk and Organize | Chunking information to alleviate working memory load combined with deliberate organization of thought. | Step-by-step instructions for chunking information by similar timeframe/ideas. Distillation of the information into an organized chronological outline, using the text from session 1 (pared down after inhibit and select exercises). Planning the processing of new information is included to promote goal oriented behavior. Information regarding working memory efficiency and thought organization are specifically integrated in the instruction. |
| 3 | Inference and Paraphrase | Previously acquired knowledge is used to understand unfamiliar/novel  information advancing the ability to restate details using college-level paraphrase skills. | Step-by-step instructions to use world knowledge to understand novel ideas paired with stepwise instruction for creating innovative approaches for communicating student understanding of materials via high-level paraphrases. |
| 4 and 5 | Synthesis | Combine details from a chunk of information into a one - two sentence synopsis of a chunk. | Examples and instruction for synthesizing while paraphrasing (see session 3) details from a chunk (see session 2) to promote optimization of working memory resources. |
| 6 | Interpretation Through Abstraction | Abstraction of underlying meanings to formulate high-level interpretative statements that have universal application. Cognitive flexibility is enhanced through deliberate reflection of various perspectives. | Questioning designed to enhance abstraction of underlying concepts, leads to thinking beyond stated details in order to produce multiple interpretations. Abstracted concepts facilitate production of global “take-away” statements that apply to a variety of narratives or experiences. Students learn to think beyond explicitly stated information by making connections between world knowledge and new information through interpretation. |
| 7 | Abstraction & Analysis | Analysis of deeper meanings specific to the presented materials, promotes abstraction and analysis. | Queries that promote pondering ideas/emotions/motives etc. in order to abstract concepts that are not directly stated. Deeper meanings are synthesized with important details to demonstrate analysis and depth of understanding. |
| 8, 9, 10 | Application and Extension | Apply and extend the cognitive processes to various forms of information, including media, video, text, and core content (literature, science, math, history). | Practice applying executive function processes to delve deeper into a variety of subjects/content; combining all cognitive processes to select, organize, synthesize, analyze and interpret deeper meanings. Students are encouraged to develop personal questions to spark abstraction and depth of thinking. |

.
